# Determinants of food insecurity among households with children in Villa el Salvador, Lima, Peru: the role of gender and employment, a cross-sectional study

**DOI:** 10.1186/s12889-022-12889-4

**Published:** 2022-04-11

**Authors:** M. Patrizia Santos, Jessica D. Brewer, Miguel A. Lopez, Valerie A. Paz-Soldan, M. Pia Chaparro

**Affiliations:** 1 Department of Epidemiology Tulane University School of Public Health and Tropical Medicine, 1440 Canal Street suite 2000, New Orleans, LA 70112 USA; 2 Department of Social, Behavioral and Population Sciences, Tulane University School of Public Health and Tropical Medicine, 1440 Canal Street suite 2210, New Orleans, LA 70112 USA; 3 Department of International Health and Sustainable Development, Tulane University School of Public Health and Tropical Medicine, 1440 Canal Street suite 2210, New Orleans, LA 70112 USA; 4 Asociación Benéfica PRISMA, Avenida Santo Toribio 115, 5to piso, San Isidro 15073 Lima, Peru

**Keywords:** Household food insecurity, Peru, Gender Equity

## Abstract

**Background:**

From 2014–2019, Latin America and the Caribbean had the fastest growth of moderate-to-severe food insecurity than any other region, rising from 22.9% to 31.7%. While the prevalence of food insecurity is higher among women than men in every continent, Latin America has the largest food insecurity gender gap. Factors contributing to this gender inequity include underrepresentation of women in formal employment, heightened burden of dependent care on women, and unequal compensation of labor for women vs. men. The objective of this study was to investigate the association between the gender of the head of the household, employment status of household members, and food insecurity in households with children in a low-income district of Lima, Peru.

**Methods:**

This cross-sectional study was carried out in Villa El Salvador, the fifth largest district in Metropolitan Lima, Peru, where over 20% of the population lives in poverty. Data were collected on a stratified random sample (*n* = 329) using a household questionnaire, including a validated food security tool (HFIAS). We ran multivariate logistic regression models predicting household food insecurity, with independent variables including gender of household head, education of household head, employment of household head, household-level employment status, age, and weekly food expenses per person.

**Results:**

In fully adjusted models, woman-headed households had almost thrice the odds of being food insecure compared to man-headed households. Education also had a significant effect size: a household whose household head did not complete high school was 3.4 times more likely to be food insecure than if they had some post-secondary education. Woman-headed households had a significantly higher proportion of members not formally employed, compared to man-headed households, but employment status was not associated with food insecurity.

**Conclusions:**

Gender of the household head was a major contributing factor to household food insecurity in Villa el Salvador. Gender dynamics affecting opportunities for employment, education, and non-remunerated work should inform national food security policies and interventions with the goal to not only lower food insecurity, but also reduce gender inequities in food insecurity and other nutritional outcomes.

**Supplementary Information:**

The online version contains supplementary material available at 10.1186/s12889-022-12889-4.

## Background

More than 820 million people in the world are hungry and approximately 2 billion are moderately or severely food insecure [1]. Food insecurity is the inability to obtain nutritious and sufficient food due to factors such as lack of monetary resources, food access, and food availability, increasing the risk of malnutrition and poor health [2]. Estimates of food insecurity reveal an upward trend in the number of people suffering from severe food insecurity worldwide, now magnified with the poverty exacerbation, food access decline, and mobility restrictions– which have limited access to work and to formal and informal food assistance resources – associated with the COVID-19 pandemic [1–3].

From 2014–2019, Latin America and the Caribbean had the fastest growth of moderate to severe food insecurity compared to any other region worldwide, rising from 22.9% to 31.7% [1]. Given this rise, exploring factors related to moderate to severe food insecurity in Latin America is warranted. While the prevalence of food insecurity is slightly higher among women than men in every continent, Latin America has the largest food insecurity gender gap, with 32.4% of women and 25.7% of men being moderately to severely food insecure in 2019 [4]. Factors contributing to this gender inequity include underrepresentation of women in formal employment, heightened burden of the care for dependents on women, and unequal compensation of labor for women vs. men [1–8]. These limitations for women may influence household food security status, where woman-headed households may be more vulnerable to be food insecure. For example, woman-headed households are more likely to live in poverty than man-headed households in Latin America [6].

There is a limited number of studies assessing the impact of the gender of the head of the household on food insecurity in Latin America; most existing studies have been conducted in the United States, Africa, and Asia [7–10]. One study conducted in Brazil reported that woman-headed households were 32% more likely to be food insecure than man-headed households [11]. However, this study did not use a validated tool to assess food insecurity. A few studies have investigated household food insecurity and gender, using a previously validated tool, in Colombia, Honduras, and Brazil [12–14]; these studies suggest that employment might be a modifying factor in the burden of food insecurity on woman-headed households. Given the high burden of food insecurity in Latin America and the Caribbean, and the potential role of inequitable employment status among women, the objective of this study was to explore the association between the gender of the head of the household, the employment status of household members, and food insecurity in a low-income district of Lima, Peru.

## Methods

### Study design and setting

This cross-sectional study was carried out in Villa El Salvador, the fifth largest district in Metropolitan Lima, Peru, where over 20% of the population lives in poverty [15]. Lima, the capital of Peru, is a highly densely populated coastal city with a population of 8,574,974 inhabitants, of which 13.1% were considered poor according to national governmental guidelines [16]. A total of 450 neighborhood blocks of Villa El Salvador from three income strata (low income, lower middle income, and middle income – 150 from each), were randomly selected, using the Peruvian National Institute of Statistics and Informatics (INEI, for its name in Spanish) database of Lima’s stratified city blocks [16]. INEI defines neighborhood block income by estimating average income per capita per household, a method detailed in Elbers et al. [17]. Our goal was to collect one household questionnaire from each of the 450 randomly selected neighborhood blocks. The eligibility criteria for the household questionnaire were the respondent had to be at least 18 years old, in charge of purchasing food for the household, and living in a household with at least one minor. While food insecurity can be experienced by households without children, this paper is part of a larger study focused on food insecurity and the role of food assistance programs in alleviating it [18]; the focus on households with children is based on the fact that most food assistance programs in Peru target minors.

Data were collected between June and July 2019 by trained staff. During this time, 329 households were surveyed. For study recruitment, the first attempt was made by knocking on the house in the most northeast corner of the randomly selected city block. If the household did not respond, refused to participate, or did not meet the eligibility criteria, the house immediately to the right was asked to participate, followed by the house immediately to the left of the first attempted house, continuing with the houses to the left until a questionnaire was completed or all the houses in the city block were approached. We knocked on the doors of 2,267 households; of these, 776 (34.2%) households did not answer the door. Of the 1,491 households that answered the door, 777 (52%) were not eligible and 385 (25.8%) refused to participate. Sampling resulted in 329 (22% of total doors knocked) completed surveys, providing a cooperation rate of eligible households of 46.1%.

### Survey instrument

The household questionnaire (additional file 1) included demographic and socioeconomic questions, items on frequency and location of food purchases, food expenses, formal and informal methods of food access, information on participation in food assistance programs, and the Household Food Insecurity Access Scale, *HFIAS* [19], which has been previously translated to Spanish and validated in Peru [20]. HFIAS asks about the frequency of specific experiences related to food insecurity, such as going to bed hungry due to lack of resources, based on the previous 30 days [20]. The questionnaire used in the study was piloted in two phases with 14 and then 5 conveniently sampled households, to validate the language and understanding of the questions [15]. A copy of the questionnaire is available upon request.

### Outcome

Our main outcome of interest was food insecurity. From the responses to HFIAS, a continuous score ranging from 0 to 27 is generated. Based on this score, households can be classified as being food secure, mildly food insecure, moderately food insecure, or severely food insecure [21]. For some analyses, the mild, moderate, and severe food insecurity categories were combined into one for a food insecurity dichotomous variable (yes/no).

### Predictor

Our main predictor was the gender of the household head. For this study, we defined household head as the survey respondent, screened to be the household member in charge of food purchasing. As this is a food insecurity study, the responsibility of acquiring food was considered the most important decision in the household.

### Covariates

Educational level of the household head was categorized as less than high school education, completed high school education, and post-secondary education. Employment of the household head was operationalized as either not-employed, self-employed, or formally employed. Self-employed categories only include renumerated work, with employment offering non-monetary compensation (e.g., piecemeal) included in this category. Moreover, household-level employment was operationalized by the proportion of household members formally employed or self-employed (categories: < 50% of household members formally employed or self-employed, 50% of household members formally employed or self-employed, and > 50% of household members formally employed or self-employed); cut-off points were chosen based on the univariate distributions in our sample. Weekly household food expenditure per person as a continuous variable was used, measured in Peruvian soles (the exchange rate was ~ 3.30 Peruvian soles per US dollar at the time of the study). Age of the household head was also considered as a covariate.

### Statistical analysis

Descriptive statistics were estimated using SAS version 9.4 (SAS Institute, Cary, NC). A chi-square test of independence was conducted to explore the bivariate associations between food insecurity and gender of the household head, and food insecurity and the other covariates. The relationship between gender of the household head and household-level employment was assessed through a likelihood ratio chi-square test.

To address multicollinearity between employment of the household head and household-level employment, we ran two separate multivariate logistic regression models predicting household food insecurity: Model 1 included gender of the household head (reference = man), employment of the household head (reference = not formally employed), age (continuous) and education of the household head (reference = less-than-high school education), and weekly household food expenditure per person. Model 2 included gender of the household head (reference = man), household-level employment (reference =  > 50% formally employed or self-employed), age (continuous) and education of the household head (reference = less-than-high school education), and weekly household food expenditure per person. An interaction term between gender and employment of household head was tested to assess moderation; the interaction term was found not significant (*p* > 0.05) and thus removed from the final model for parsimony.

## Results

Of the 329 households surveyed, 254 (77.2%) were classified as food insecure, with 49 (19.3%) identified as mildly food insecure, 79 (31.1%) as moderately food insecure, and 126 (49.6%) as severely food insecure. Therefore, the minority group was the food secure, which represented only 22.8% (*n* = 75) of the sample. Demographic household characteristics are presented in Table 1.

**Table 1:** Sociodemographic characteristics of included households from Villa El Salvador (Peru), by food security status (*N* = 329)

**Variables**	**Measurements**	**Descriptive statistics by food security status**			
		**Food secure**	**Mildly Food insecure**	**Moderately food insecure**	**Severely food insecure**
		**Mean (St. Dev.)**			
Age	Years	39.31(12.37)	36.96 (11.24)	37.35 (12.31)	44.16 (13.06)
Weekly household food per person	Peruvian soles	43.04 (19.97)	37.92 (14.90)	36.42 (13.52)	30.84 (14.35)
		**Frequency (%)**			
Gender	Women	64 (85.33)	45 (91.84)	77 (97.47)	117 (92.86)
Education	< High school graduate	11 (14.67)	7 (14.29)	21 (26.58)	58 (46.03)
	High school graduate	24 (32.00)	23 (46.94)	28 (35.44)	40 (31.75)
	Some college or technical school	40 (53.33)	19 (38.78)	30 (37.97)	28 (22.22)
Employment of household head	Not employed	44 (58.67)	35 (69.39)	52 (65.82)	84 (66.67)
	Self-employed	23 (30.67)	10 (20.41)	17 (21.52)	29 (23.02)
	Formally employed	8 (10.67)	5 (10.20)	10 (13.25)	13 (10.32)
Household level employment status^a^	< 50% of household members employed	10 (13.33)	10 (20.41)	16 (20.25)	22 (17.46)
	50% of household members employed	25 (33.33)	16 (32.65)	29 (36.71)	45 (35.71)
	> 50% of household members employed	40 (53.33)	23 (46.94)	34 (43.04)	59 (46.83)
Neighborhood income strata	Lower income block	19 (18.27)	14 (13.46)	26 (25.00)	45 (43.27)
	Lower-middle income block	29 (23.97)	21 (17.36)	28 (23.14)	43 (35.54)
	Middle income block	27 (25.96)	14 (13.46)	25 (24.04)	38 (36.54)

^a^Household-level employment status includes those formally employed and self-employed

Note: Neighborhood income percentages is calculated by row, not column

The proportion of women-headed households increased with increased food insecurity severity. The mean age of the head of the household also increased with severity of food insecurity (mean age in years [SD]: food secure = 39.3 [12.4], mildly food insecure = 37.0 [11.2], moderately food insecure = 37.4 [12.3], severely food insecure = 44.2 [13.1]), while weekly household food expenditures per person decreased with increased food insecurity severity (mean in Peruvian soles [SD]: food secure = 43.0 [20.0], mildly food insecure = 37.9 [14.9], moderately food insecure = 36.4 [13.5], severely food insecure = 30.8 [14.4]). Regardless of food insecurity severity, the weekly household food expenditure per person in the sample was lower than the cost of the basic food basket in Peru, which was 46 soles in 2019; the basic food basket is defined as one including the most inexpensive foods a person or household can buy to meet the minimum daily food consumption to satisfy nutritional requirements [21]. Among food insecure household heads, 34% had less than high school educational attainment, 36% completed high school, and 30% had at least some college or technical school. More than two-thirds of food insecure household heads were not formally employed, 22% were self-employed, and only 11% were formally employed. The chi-square test of independence (*p*-value = 0.0207) provided evidence that gender of household head and household food insecurity were significantly associated.

Compared to man-headed households, woman-headed households had a significantly higher proportion of households where more than half of the members were not formally or self-employed (data not shown; *p*-value = 0.0330). Only 13.6% of woman-headed households had more than half of its members formally employed, compared to 30.8% in the man-headed households. In terms of formal employment, 41% of woman-headed households had zero of their members formally employed, compared to 23% of man-headed households.

Table 2 displays the results of the multivariable logistic regression models. In Model 1, woman-headed households had 2.8 times the odds of being food insecure compared to man-headed households, adjusting for age, education, and employment of the household head, and household food expenditure per person. Education had a significant association with food insecurity: compared to households with a household head who had some post-secondary education, households with household heads who did not complete high school and households with household heads with a high school education were 3.4 times and 2.3 times more likely, respectively, to be food insecure. Employment of the household head was not associated with food insecurity.

**Table 2:** Results of the multivariable logistic regression models predicting household food insecurity by gender of household head

	**Model 1**	**Model 2**
	**OR (95% CI)**	**OR (95% CI)**
Man vs. Woman Household Head^a^	2.81 (1.06, 7.45)	2.58 (1.05, 6.38)
**Employment of Household Head**		
Not employed vs. Formally employed^a^	1.64 (0.64, 4.21)	
Self-employed vs. Formally employed^a^	0.99 (0.49, 2.00)	
**Household-level employment status**		
< 50% vs. > 50% of household members employed^a^		1.26 (0.56, 2.85)
50% vs > 50% of household members employed^a^		1.16 (0.63, 2.15)
< 50% vs 50% of household members employed^a^		1.08 (0.46, 2.55)
**Age of Household Head (continuous)**	1.00 (0.98, 1.03)	1.00 (0.98, 1.03)
**Education of Household Head**		
< High school graduate vs. ≥ Some college or technical school^a^	3.37 (1.54, 7.39)	3.18 (1.46, 6.94)
High school graduate vs. ≥ Some college or technical school^a^	2.25 (1.20, 4.21)	2.26 (1.17, 4.39)
**Weekly Food Expenses per Person (continuous)**	0.97 (0.96, 0.99)	0.97 (0.96, 0.99)
Hosmer and Lemeshow Goodness-of-Fit Test	χ^2^ = 4.06 df = 8 *p*-value = 0.85	χ^2^ = 4.84 df = 8 *p*-value = 0.77

^a^Indicated reference group

Similarly, in Model 2 (Table 2), woman-headed households had 2.6 times the odds of being food insecure compared to man-headed households, adjusting for age and education of the household head, household-level employment, and household food expenditure per person. Education was again significantly associated with food insecurity and household-level employment status was not associated with food insecurity.

## Discussion

In this study based on households with children living in a low-income district in Lima, Peru, we found that gender of the household head – defined as the person in charge of food purchases – is an important determinant of household food insecurity. Similar to what previous studies in Colombia, Honduras, and Brazil have reported [12–14], we found that woman-headed households are at higher risk of food insecurity. Woman-headed households in our sample had a higher proportion of members not formally employed or self-employed in their homes; however, we found no association between employment status of the household head and household food insecurity. Gender disparities in type of employment were found to be associated with money shortage, a proxy indicator for food insecurity, in a study using data from Ecuador, Bolivia, Thailand, and the Philippines, with women in households with money shortages being more likely to be self-employed, as opposed to being salaried employees, when compared to women in households without money shortages [22]. This multi-country study used an indirect measure of food insecurity, contrary to our study which used HFIAS, a validated tool to measure food insecurity, which may explain the different results.

Given the disproportionate burden of food insecurity that falls on woman-headed households, strategies to overcome food insecurity should aim to 1) combat the social determinants that make women more vulnerable to food-specific poverty such as access to education, and 2) incorporate interventions aimed to influence gender dynamics/roles into national food security policies, including implementing efforts to reduce their non-income producing workload within the household [22]. Regardless of employment status, men earn more than women, on average, largely because women are more often employed in the informal sector of the economy in Latin America [23–25], furthering women’s disadvantage. Moreover, women are often responsible for completing domestic tasks, limiting the number of hours they can use to generate income [23, 25]. In Peru, specifically, men earn on average 45% more than women; 6% of this wage gap is explained by individual characteristics (e.g., differential educational attainments), 11% by differences in supports (i.e., individual characteristics of men and women being distributed differently impeding appropriate matching), and 28% by non-observable individual characteristics, among which we can include social roles [26]. The burden women carry – through ascribed social norms regarding the type of work they do and household and childcare responsibilities – can reduce the income generating opportunities for woman-headed households [27, 28]. Previous interventions that have positively influenced gender dynamics within the household include in-person and video outreach to husbands and mothers-in-law, and women's support groups on financial and gender issues [29, 30].

This study has strengths and limitations. Strengths include cluster random sampling of households, the exploration of the impact of not only household headship but household composition, and the use of a validated tool to measure the outcome of food insecurity. Additionally, by defining household headship as the individual who oversees the household’s food purchasing, we were able to explore the importance and impact of gendered tasks that do not generate income. As for limitations, volunteer bias could have affected our results given our low response rate and generalizability of our results might be limited to other similar urban poor areas of Peru. Furthermore, it is possible that men answered our household questionnaire differently than women – especially the food insecurity questions – which may partly explain our results. Men might present higher social desirability bias while responding, as in a patriarchal society it might seem especially socially undesirable to be perceived to have failed to provide food for one’s family as a man [31]. Additionally, the number of man-headed households in our sample is significantly smaller than that of woman-headed households, affecting their comparability in terms of distribution by food insecurity severity.

Having a man-headed household in terms of overseeing food shopping could also be an indicator of household dynamics, such as a more liberal household in terms of not following gender roles. For example, a study using data from Mexico, Peru, and Ecuador found that countries with more biased gender social norms had women spend more time in unpaid tasks such as food shopping [32]. Previous studies have also found an association between women’s decision-making ability over household purchases (including food) and their age, employment, and number of dependents in households, all factors that have also been associated with household food insecurity [33]. While our screening question prior to initiating the household questionnaire explicitly asked for the person “in charge” of food purchasing, we cannot conclude to what extent those decisions are autonomous or driven by other household members. Given the high prevalence of informal employment in the study area, we did not ask participants about household income. While we controlled for food expenditures, the same expenditure amount could have very different implications for households at higher vs. lower income levels. Finally, our questionnaire did not allow to compare those without formal employment that are actively seeking and those that are not actively seeking this type of employment.

Food security is an intersectional issue related to income and poverty, access to productive resources, and gender-based discrimination [33]. Therefore, to ensure the reduction of food insecurity it is essential to integrate a gender equity perspective at all stages and levels of policies, programs and projects aiming to combat food insecurity paying particular attention to the social determinants of health (e.g., educational attainment) that intersect with gender [33, 34]. While we recognize this is a long-term effort, less resource- and time-intensive efforts to influence gender norms with the aim to decrease the unique burden on women to do unpaid care and domestic work can be implemented. These projects to influence gender norms must use interactive approaches to foster reflection, messages should be shared regularly and through a range of channels (e.g., social media, TV, radio, etc.), members and role models from the community must be called-in to participate in the program, and the program must engage men, while recognizing that big changes happen in small steps [35].

## Conclusion

Women-headed households were found to be at higher risk of food insecurity compared to men-headed households in a low-income district in Lima, Peru. The gender dynamics affecting opportunities for remunerated employment and education for women should inform national food security policies and interventions with the goal of not only lowering food insecurity in the country, but also reducing gender inequities in food insecurity and other nutritional outcomes. Simultaneously, efforts should be placed in interventions addressing gender norms with the goal of reducing gendered workloads within the household and increasing women’s engagement in income producing activities.

## Supplementary Information

**Additional file 1.**

## Data Availability

The datasets used and/or analyzed during the current study are available from the corresponding author on reasonable request.

## Abbreviation

HFIAS: Household Food Insecurity Access Scale
