# Supplementary material for: Determinants of food insecurity among households with children in Villa el Salvador, Lima, Peru: the role of gender and employment, a cross-sectional study
Source: BMC Public Health. 2022 Apr 11;22:717. doi: 10.1186/s12889-022-12889-4 (PMC8996213; doi:10.1186/s12889-022-12889-4)
Supplement: Supplementary file 1 — Additional file 1. [file 12889_2022_12889_MOESM1_ESM.docx]

**Food Security Survey:**

**Project:** Type of food assistance used by families with food insecurity in Lima, Peru

**Collaborating Institutions:** Asociación Benéfica PRISMA and Tulane University

**Principal Investigator:** Dr. Maria Pia Chaparro

Date: ___/____/____ Start time: ___:___ AM/PM End time: ___:___AM/PM

Interviewer: ___________________________ Participant code: ____________________

Interview district: ______ Neighborhood block code: _________________________________

GPS location of the house: _______________________________________________

**Eligibility:**

**We are going to start with a few questions about you and your household.**

1. In your household, does there live any minor under the age of 18?

Yes………………………………………………….1

No…… NOT ELIGIBLE……………..2

1. Are you in charge of purchasing the food for your household?

Yes………. GO TO QUESTION 4……….1

No………………………………………..…………..2

1. Can we speak with the person in charge of purchasing the food for your household?

Yes……………BEGIN THE INTERVIEW WITH THAT PERSON…………………………..………1

No…….NOT ELIGIBLE……………2

1. What is your age, please?

____________________________ Years old

Interviewee is 18 years or older………………………………………….…………1

Interviewee is less than 18 years old…………..NOT ELIGIBLE………….2

**PART I. Demographics and Socioeconomic Variables**

1. [ASK ONLY IF YOU ARE UNSURE] Are you of the masculine or feminine sex?

Man………………………………..1

Woman……………………………………2

Other……………………………………..3

1. What is your current marital status?

Single…………………………….1

Cohabiting partner…………………………2

Married……………..……………..3

Separated………..……………….4

Divorced……….………………5

Widowed…………….………………..6

1. What is the highest level of education you have achieved?

No schooling………………………………1

Early education…………………………..2

Some primary school………………………3

Completed primary school………………………...4

Some secondary school………………….5

Completed secondary school…………………….6

Some technical training…………..7

Completed technical training……………..8

Some university….9

Completed university…….10

1. Currently, what is your primary occupation?

[WRITE THE EXACT RESPONSE, THEN MARK THE CATEGORY]

__________________________________________________________Exact response

Public sector employee…………1

Private sector employee………..2

Self-employed/merchant……………….3

Student………………………………………………………………..4

Homemaker…………………………………………………………5

Unemployed……………………………………………………………….6

Retired……………………………………………………………….7

1. How many people live in this household?

______________________Number of people

1. Of the [X NUMBER OF PEOPLE FROM QUESTION 5] people who live here, do they share the same budget and share a common pot of food?

Yes………….GO TO QUESTION 7…..1

No…………………………………………………….2

1. How many people, of those who live in the household, share the same budget and share a common pot of food?

_______________________Number of people

1. I am going to ask you some questions about these [X NUMBER FROM QUESTION 5 OR 7] people apart from yourself:

|  | a. What is this person’s name? | b. What is this person’s relationship to you?  Wife/husband, or partner……1  Child…2  Mother/father…3  Stepchild..4  Son-in-law or daughter-in-law…5  Grandchild…6  Sibling…7  Brother-in-law or sister-in-law…8  Other relative…9  Other, not relative...10 | c. How old is [NAME]?  Less than 1 year……00  Age unknown, less than 18 years…98  Age unknown, 18 years or more…99 | d. Is [NAME] of the masculine or feminine sex?  Man...1  Woman…2  Other…3 | e. What is the highest level of education [NAME] has achieved?  [IF CURRENTLY STUDYING]  What is their current level of education?  No schooling…1  Hasn’t entered school…2  Early education…3  Some primary school…4  Completed primary school…5  Some secondary school…6  Completed secondary school…7  Some technical training…8  Completed technical training…9  Some university…10  Completed university…11 | f. What is [NAME]’s primary occupation currently?  Public sector employee…1  Private sector employee...2  Self-employed/ merchant…3  Student…4  Homemaker…5  Unemployed…6  Retired…7 |
| --- | --- | --- | --- | --- | --- | --- |
|  | WRITE NAME | WRITE CODE | WRITE EXACT AGE IN YEARS OR WRITE CODE | WRITE CODE | WRITE CODE | WRITE THE COMPLETE ANSWER AND WRITE CODE |
| 1 |  |  |  |  |  |  |
| 2 |  |  |  |  |  |  |
| 3 |  |  |  |  |  |  |
| 4 |  |  |  |  |  |  |
| 5 |  |  |  |  |  |  |
| 6 |  |  |  |  |  |  |
| 7 |  |  |  |  |  |  |

**Now, I am going to ask you about the amenities you have in your home.**

1. What amenities do you have in this house/home?
   1. Electricity?

Yes……………………………1

No………………………….2

- 1. Landline phone?

Yes…………………………..1

No………………………..2

- 1. Cell phone?

Yes…………………………….……1

No……GO TO PART E….2

- 1. Internet access by cell phone?

Yes………………………………….1

No………………………………..2

- 1. Internet?

Yes……………………………….….1

No…………………….…………..2

- 1. Cable television?

Yes……………………………….….1

No…………………………………2

1. I’m going to read you a list of household appliances and vehicles that you could have in your house. Please, for each one, tell me if you have it in your house, if it is working, and how many you have of each one.

[READ EACH ITEM AND WRITE THE QUANTITY OF EACH ONE THAT IS IN USE (NOT BROKEN). IF THEY DO NOT HAVE IT, WRITE 0.]

| Appliance | # | Appliance | # | Vehicle | # |
| --- | --- | --- | --- | --- | --- |
| Refrigerator |  | Radio/sound system |  | Bicycle/tricycle |  |
| Television |  | Electric or gas stove |  | Motorcycle |  |
| Blender |  | Washer |  | Motorcycle car/motorcycle taxi |  |
| Computer/Laptop/Tablet |  | Microwave |  | Car |  |
| Fan |  | Other- DESCRIBE: | | |  |

1. Do you have some form of health insurance?

Yes……………………………………………….1

No…….GO TO PART II…….2

1. What type of insurance do you have?

SIS (public health insurance for the needy)…………….1

*EsSalud* (public health insurance for formally employed people)………………………….2

Police/Military Insurance……………..3

Private insurance……………………..4

Other…………………………………………5

DESCRIBE: _______________________________________________________

Does not know……………………………………6

**PART II. Household Food Insecurity Access Scale:**

**I am going to ask you some questions about your experiences in terms of the foods that are consumed in your household and the resources you have to be able to purchase these foods. When answering these questions, please focus on the [X NUMBER OF PEOPLE FROM QUESTION 5 OR 7] people in your household with whom you share the same budget and a common pot of food.**

1. In the past 4 weeks, did you or anyone household member worry that your household would not have enough food?

Yes…………………………………………….1

No……..GO TO QUESTION 14……..2

13a. How frequently did this occur?

Rarely (1 or 2 times)…………………1

Sometimes (3-10 times)…………..2

Often (more than 10 times)……..3

1. In the past 4 weeks, were you or any household member not able to eat the kinds of foods you preferred because of a lack of resources?

Yes……………………………………………1

No………GO TO QUESTION 15……2

14a. How frequently did this occur?

Rarely (1 or 2 times)…………………1

Sometimes (3-10 times)…………..2

Often (more than 10 times)……..3

1. In the past 4 weeks, did you or any household member have to eat a limited variety of foods due to a lack of resources?

Yes…………………………………………..1

No………GO TO QUESTION 16……2

15a. How frequently did this occur?

Rarely (1 or 2 times)…………………1

Sometimes (3-10 times)…………..2

Often (more than 10 times)……..3

1. In the past 4 weeks, did you or any household member have to eat some foods that you really did not want to eat because of a lack of resources to obtain other types of foods?

Yes……………………………………………1

No………GO TO QUESTION 17…….2

16a. How frequently did this occur?

Rarely (1 or 2 times)…………………1

Sometimes (3-10 times)…………..2

Often (more than 10 times)……..3

1. In the past 4 weeks, did you or any other household member have to eat a smaller meal than you felt you needed because there was not enough food?

Yes……………………………………………1

No……GO TO QUESTION 18………2

17a. How frequently did this occur?

Rarely (1 or 2 times)…………………1

Sometimes (3-10 times)…………..2

Often (more than 10 times)……..3

1. In the past 4 weeks, did you or any household member have to eat fewer meals in a day because there was not enough food?

Yes……………………………………………1

No……GO TO QUESTION 19………2

18a. How frequently did this occur?

Rarely (1 or 2 times)…………………1

Sometimes (3-10 times)…………..2

Often (more than 10 times)……..3

1. In the past 4 weeks, was there ever no food to eat of any kind in your household because of lack of resources to get food?

Yes……………………………………………1

No……GO TO QUESTION 20………1

19a. How frequently did this occur?

Rarely (1 or 2 times)…………………1

Sometimes (3-10 times)…………..2

Often (more than 10 times)……..3

1. In the past 4 weeks, did you or any household member go to sleep at night hungry because there was not enough food?

Yes…………………………………………..1

No……GO TO QUESTION 21………2

20a. How frequently did this occur?

Rarely (1 or 2 times)…………………1

Sometimes (3-10 times)…………..2

Often (more than 10 times)……..3

1. In the past 4 weeks, did you or any household member go a whole day and night without eating anything because there was not enough food?

Yes…………………………………………….1

No……GO TO PART III…………………2

21a. How frequently did this occur?

Rarely (1 or 2 times)…………………1

Sometimes (3-10 times)…………..2

Often (more than 10 times)……..3

**PART III. Food Assistance Program Participation and Informal Methods of Food Access**

**Now I am going to ask you about food assistance programs and your participation in them.**

1. Is there a *Comedor Popular* (communal kitchen) in your neighborhood?

Yes…………………………………………….….1

No…..GO TO QUESTION 33….2

1. How many *Comedores Populares* are there in your neighborhood?

______________________________Number of *Comedores Populares*

1. Where is/are this/these *Comedor Popular*?

[ASK FOR THE ADDRESS IF POSSIBLE]

______________________________

1. Have you ever used the *Comedor Popular* in your neighborhood?

Yes………………………………………1

No……GO TO QUESTION 32….2

1. When was the last time you used the Comedor Popular?

[WRITE THE COMPLETE ANSWER AND WRITE THE CODE]: _______________________

In the last 4 weeks………………………………………………….1

In the last 3 months………………………………………………..2

In the last 6 months…………………………………………………3

More than 6 months ago…….GO TO QUESTION 33….4

1. How many times a week do you go to the *Comedor Popular* in your neighborhood?

______________________________Number of times

1. How many *Comedores Populares* do you go to?

____________________________Number of *Comedores Populares*

1. Where are all of the *Comedores Populares* that you go to?

[ASK FOR THE ADDRESS IF POSSIBLE]

____________________________________________________________________

1. What is the main reason you go to the *Comedor Popular*?

[WRITE THE COMPLETE ANSWER]: _________________________________________

________________________________________________________________________

________________________________________________________________________

1. When you go the *Comedor Popular*, the serving that you purchase, do you share it with your relatives or is it just for your personal consumption?

Shares with relatives………………………..1

For personal consumption…………………………2

[GO TO QUESTION 33]

1. What is the main reason you don’t go to the *Comedor*?

WRITE THE COMPLETE ANSWER: __________________________________________

________________________________________________________________________

________________________________________________________________________

1. [FOR HOUSEHOLD MEMBERS WHO ARE LESS THAN 18 YEARS OLD]

|  | a. Name and Age of Child | b. Has [CHILD’S NAME] received *Vaso de Leche* (daily food ration)?  Yes, in the past 4 weeks …..1  Yes, in the past 3 months……2  Yes, in the past 6 months……3  No…GO TO PART F….4 | c. How many times a week does [CHILD’S NAME] receive *Vaso de Leche*? | d. Does [CHILD’S NAME] eat all of the food they’re given in *Vaso de Leche*?  Yes, they eat it on their own…1  Yes, they eat it and share it with others…2  No…3  Other…4  [DESCRIBE] | e. What is the main reason [CHILD’S NAME] eats *Vaso de Leche*?  [GO TO PART G] | f. What is the main reason [CHILD’S NAME] does not eat *Vaso de Leche*? | g. [IF THE CHILD IS LESS THAN 3 YEARS OLD] Does [CHILD’S NAME] attend *Cuna Más* (public daycare)?  Yes…1  No…GO TO PART O…2 | h. Has [CHILD’S NAME] eaten food in *Cuna Más*?  Yes, in the past 4 weeks…1  Yes, in the past 3 months…2  Yes, in the past 6 months…3  No…GO TO PART N….4 | i. How many times a week does [CHILD’S NAME] eat food in *Cuna Más*? | j. How many meals and snacks a day does [CHILD’S NAME] eat in *Cuna Más*? | k. Does [CHILD’S NAME] eat all of the food they’re given in *Cuna Más*?  Yes, they eat it in Cuna Más…1  Yes, they eat it on their own at home…2  Yes, they eat it at home and share it with others…3  No…4  Other…5  [DESCRIBE] |
| --- | --- | --- | --- | --- | --- | --- | --- | --- | --- | --- | --- |
|  | COPY FROM QUESTION 8 |  | WRITE THE NUMBER OF TIMES PER WEEK |  | WRITE THE COMPLETE ANSWER | WRITE THE COMPLETE ANSWER |  |  | WRITE THE NUMBER OF TIMES PER WEEK | WRITE THE NUMBER OF MEALS AND SNACKS PER DAY |  |
| 1 |  |  |  |  |  |  |  |  |  |  |  |
| 2 |  |  |  |  |  |  |  |  |  |  |  |
| 3 |  |  |  |  |  |  |  |  |  |  |  |
| 4 |  |  |  |  |  |  |  |  |  |  |  |
| 5 |  |  |  |  |  |  |  |  |  |  |  |

33. (Continued)

|  | l. What type of foods do they offer in *Cuna Más*? | m. What is the main reason [CHILD’S NAME] eats food in *Cuna Más*?  [GO TO PART O] | n. What is the main reason [CHILD’S NAME] does not eat food in *Cuna Más*? | o. [IF THE CHILD IS 6-18 YEARS OLD] Does [CHILD’S NAME]’s school participate in *Qali Warma*/ *Desayuno Escolar* (school breakfast)?  Yes…1  No…GO TO PART A FOR THE NEXT HOUSEHOLD MEMBER OR QUESTION 34...2 | p. Has [CHILD’S NAME] received foods from *Qali Warma*?  Yes, in the past 4 weeks …1  Yes, in the past 3 months…2  Yes, in the past 6 months…3  No…GO TO PART U…4 | q. How many times a week does [CHILD’S NAME] receive foods from *Qali Warma*? | r. Does [CHILD’S NAME] eat all of the food they’re given in *Qali Warma*?  Yes, they eat it at school…1  Yes, they eat it on their own at home…2  Yes, they eat it at home and share it with others…3  No…4  Other…5  [DESCRIBE] | s. What type of foods do they offer in *Qali Warma*? | t. What is the main reason [CHILD’S NAME] eats foods from *Qali Warma*?  [GO TO PART A FOR THE NEXT HOUSEHOLD MEMBER OR QUESTION 34] | s. What is the main reason [CHILD’S NAME] does not eat food from *Qali Warma*? |
| --- | --- | --- | --- | --- | --- | --- | --- | --- | --- | --- |
|  | WRITE THE COMPLETE ANSWER OR THAT THEY DO NOT KNOW | WRITE THE COMPLETE ANSWER | WRITE THE COMPLETE ANSWER |  |  | WRITE THE NUMBER OF TIMES PER WEEK |  | WRITE THE COMPLETE ANSWER OR THAT THEY DO NOT KNOW | WRITE THE COMPLETE ANSWER | WRITE THE COMPLETE ANSWER |
| 1 |  |  |  |  |  |  |  |  |  |  |
| 2 |  |  |  |  |  |  |  |  |  |  |
| 3 |  |  |  |  |  |  |  |  |  |  |
| 4 |  |  |  |  |  |  |  |  |  |  |
| 5 |  |  |  |  |  |  |  |  |  |  |

1. Do you or any other household member participate in another food assistance program that we have not mentioned?

Yes………………………………………………….1

No……GO TO QUESTION 37………..2

1. In what program(s) do you all participate?

WRITE THE COMPLETE ANSWER: __________________________________________

________________________________________________________________________

1. Who in your household participates in these other food assistance programs?

WRITE THE COMPLETE ANSWER AND COMPARE IT TO THE HOUSEHOLD MEMBERS [QUESTION 8]: __________________________________________________________

________________________________________________________________________

**Now I am going to ask you a series of questions related to other methods you could use to get food.**

1. In the past 7 days, how much money did you spend on food (to feed the members of your household)?

________________Peruvian soles per week **O** _________________ Peruvian soles daily

1. How many people were fed with the food that you purchased in the past 7 days?

_______________ Number of people

1. In the past 7 days, did you get any food on credit (for yourself or the members of your household)?

Yes…………………………………………………….1

No……….GO TO QUESTION 44……….2

1. Where did you get this food on credit?

From a bodega…………………………………..1

From a commercial store……………………2

From a street vendor…………….…3

From a market stall………………………………..4

Other…………………………………………………..5

[DESCRIBE]: ____________________________________________

1. In the past 7 days, did you get food on credit in more than one place?

Yes…………………………………………………….1

No………………………………………….……….2

1. What was the monetary value in Peruvian soles of the food that you got on credit in the past 7 days?

_________________Peruvian soles

1. Are you worried about paying your balance before purchasing more food in this place?

Not at all worried…………………….1

Somewhat worried…………………………2

Worried……………………………..3

Very worried……………………..4

1. In the past 7 days, did you borrow money to purchase the foods in your household?

Yes…………………………………………………….1

No……….GO TO QUESTION 47…….2

1. From whom did you borrow this money principally?

From a relative………………………………1

From a neighbor…………………………..2

From a friend……………………………3

From a moneylender………………………………..4

Other……………………………………………….5

[DESCRIBE]: ________________________________________________

1. Are you worried about paying this borrowed money on time?

Not at all worried…………………….1

Somewhat worried…………………………2

Worried……………………………..3

Very worried……………………..4

1. In the past week, did you give food to someone outside of your household?

Yes…………………………………………………….1

No……….GO TO QUESTION 49……….2

1. To whom did you give this food?

CHECK ALL THAT APPLY

To relatives……….…1

To neighbors……………..2

To friends………………3

Other…………………………..5

[DESCRIBE]: _______________________________________

1. In the past 7 days, have you received free food from someone outside of your household?

Yes…………………………………………………….1

No……….GO TO QUESTION 54……….2

1. How many times in the past 7 days have you received free food from someone outside of your household?

____________________Number of times

1. From whom did you receive this free food in the past 7 days?

CHECK ALL THAT APPLY

From a relative……………………1

From a neighbor………………..2

From a friend………………..3

Other……………………………………4

[DESCRIBE]: ________________________________________

1. Did you offer something to this person in exchange for the food that they gave you?

YES…………………………………………….1

No……GO TO QUESTION 54…..2

1. What did you offer in exchange for the food?

[WRITE THE COMPLETE ANSWER]: _________________________________________

________________________________________________________________________

1. In the past 7 days, have you prepared food with someone who is not a member of your household (ex. friend, neighbor)?

Yes………………………………………..1

No…GO TO QUESTION 59…2

1. How many times in the past 7 days have you prepared food with someone who is not a member of your household?

_____________________________ Number of times

1. In the past 7 days, with how many people who are not members of your household have you prepared food?

__________________________ Number of people

1. What is this/these person’s/people’s relationship to you?

[CHECK ALL THAT APPLY]

Neighbor………………………………………1

Friend……………………………………………2

Relative…………………………………………3

Other……………………………………….……4

[DESCRIBE]: ____________________________________________________

1. In the past 7 days, where have you prepared food with someone who is not a member of your household?

[CHECK ALL THAT APPLY]

In the participant’s home…1

In the other person’s home…2

In the *Comedor Popular*……………3

Other…………………………………………4

[DESCRIBE]: ______________________________________________________

1. Do you have a garden or farm that provides food?

Yes………………………………………1

No……GO TO PART IV……….…2

1. Have you or any member of your household ever eaten foods from your garden/farm?

Yes………………………………………1

No……GO TO PART IV………….2

1. When was the last time you or any member of your household ate food from your garden/farm?

In the last 4 weeks………………………………………..1

In the last 3 months………………………………………2

In the last 6 months……………………………………..3

More than 6 months ago……GO TO PART IV…4

1. How many times a week does a member of your household eat foods from your garden/farm?

______________________________Number of times

**PART V. Perceptions of the Selection and Quality of Food Available in the Neighborhood and Characteristics of Commonly Visited Food Outlets**

**Now I am going to ask you some questions about the foods available in your neighborhood and where you purchase a majority of the food for your household.**

1. Thinking of your neighborhood as the area within about a **20-minute walk** from your home, please tell me whether you agree or disagree with the following statements:
   1. It is easy to buy fresh fruits and vegetables in your neighborhood.

Strongly disagree…………1

Somewhat disagree…………………………….2

Neither agree nor disagree…3

Somewhat agree………………………………….4

Strongly agree……………….5

- 1. The fresh fruits and vegetables in your neighborhood are of high quality.

Strongly disagree………….1

Somewhat disagree…………………………….2

Neither agree nor disagree…3

Somewhat agree………………………………….4

Strongly agree……………….5

- 1. There is a large selection of fresh fruits and vegetables in your neighborhood.

Strongly disagree………….1

Somewhat disagree…………………………….2

Neither agree nor disagree…3

Somewhat agree………………………………….4

Strongly agree……………….5

- 1. It is easy to buy low-fat products, such as low-fat milk or lean meats, in your neighborhood.

Strongly disagree………….1

Somewhat disagree…………………………….2

Neither agree nor disagree…3

Somewhat agree………………………………….4

Strongly agree……………….5

- 1. The low-fat products, such as low-fat milk or lean meats, in your neighborhood are of high quality.

Strongly disagree………….1

Somewhat disagree…………………………….2

Neither agree nor disagree…3

Somewhat agree………………………………….4

Strongly agree……………….5

- 1. There is a large selection of low-fat products, such as low-fat milk or lean meats, available in your neighborhood.

Strongly disagree………….1

Somewhat disagree…………………………….2

Neither agree nor disagree…3

Somewhat agree………………………………….4

Strongly agree……………….5

1. In how many outlets do you purchase a majority of the food that is eaten in your household?

1 store………………..1

2 stores……………….2

More than 2 stores…3

1. In what types of outlets do you purchase a majority of the food that is eaten in your household?

[CHECK ALL THAT APPLY]

Street vendor………………….1

Bodega……………………………………….2

Market…………………………………….3

Supermarket……………………………4

Restaurant……………………………….5

Other……………………………………………6

DESCRIBE: ____________________________________________

1. What are the names of the three outlets where you purchase a majority of the food that is eaten in your household?
2. ____________________________________________________
3. ____________________________________________________
4. ____________________________________________________
5. Now, I am going to ask you some questions about these 3 outlets where you purchase a majority of the food that is eaten in your household.

|  | a. Name of place | b. What type of place is [NAME OF PLACE]?  Street vendor…1  Bodega…2  Market…3  Supermarket…4  Restaurant…5  Other…6  [DESCRIBE] | c. ¿Where is [NAME OF PLACE]? | c. How frequently do you purchase food in [NAME OF PLACE]?  Every month….1  Every two weeks….2  1 time a week….3  More than 1 time a week...4  Every day….5  Other…6  [DESCRIBE] | e. Usually, how do you get to [NAME OF PLACE]?  Walking...1  By bicycle...2  By bus or other type of public transportation (Metro, train)...3  By personal car or motorcycle…4  By taxi or motorcycle taxi…5  By car/ motorcycle of an acquaintance (who takes me)…6  Other…7  [DESCRIBE] | f. If you had to **walk** from your house to [NAME OF PLACE], how many minutes would it take you?  10 minutes or less…1  11 to 20 minutes…2  21 to 30 minutes…3  More than 30 minutes…4 |
| --- | --- | --- | --- | --- | --- | --- |
|  | COPY FROM QUESTION 66 | WRITE THE CODE AND WRITE THE COMPLETE ANSWER IF IT IS “OTHER” | WRITE THE COMPLETE ANSWER AND ASK FOR THE ADDRESS |  | WRITE THE CODE AND WRITE THE COMPLETE ANSWER IF IT IS “OTHER” |  |
| 1 |  |  |  |  |  |  |
| 2 |  |  |  |  |  |  |
| 3 |  |  |  |  |  |  |

67. (Continued) Please tell me whether you agree or disagree with the following statements:

|  | g. [NAME OF PLACE] is close to your house.  Strongly disagree…1  Somewhat disagree…2  Neither agree nor disagree…3  Somewhat agree…4  Strongly agree…5 | h. [NAME OF PLACE] is near other places where you spend time, for example, a school, workplace, or relatives’ house.  Strongly disagree…1  Somewhat disagree…2  Neither agree nor disagree…3  Somewhat agree…4  Strongly agree…5 | i. Your friends/ relatives also shop at [NAME OF PLACE].  Strongly disagree…1  Somewhat disagree…2  Neither agree nor disagree…3  Somewhat agree…4  Strongly agree…5 | j. There is a wide variety of foods in [NAME OF PLACE].  Strongly disagree…1  Somewhat disagree…2  Neither agree nor disagree…3  Somewhat agree…4  Strongly agree…5 | k. There is a high quality of foods in [NAME OF PLACE].  Strongly disagree…1  Somewhat disagree…2  Neither agree nor disagree…3  Somewhat agree…4  Strongly agree…5 | l. The price of foods in [NAME OF PLACE] are accessible.  Strongly disagree…1  Somewhat disagree…2  Neither agree nor disagree…3  Somewhat agree…4  Strongly agree…5 | m. There is access to public transport near/ to arrive to [NAME OF PLACE].  Strongly disagree…1  Somewhat disagree…2  Neither agree nor disagree…3  Somewhat agree…4  Strongly agree…5 |
| --- | --- | --- | --- | --- | --- | --- | --- |
| 1 |  |  |  |  |  |  |  |
| 2 |  |  |  |  |  |  |  |
| 3 |  |  |  |  |  |  |  |

67. (Continued)

|  | n. In [NAME OF PLACE], how easy is it to get fresh fruits and vegetables?  Very easy…1  Somewhat easy…2  Somewhat hard…3  Very hard…4 | o.. In [NAME OF PLACE], how easy is it to get lean meats?  Very easy…1  Somewhat easy…2  Somewhat hard…3  Very hard…4 | p. In [NAME OF PLACE], how easy is it to get low fat products, like low-fat milk or yogurt?  Very easy…1  Somewhat easy…2  Somewhat hard…3  Very hard…4 | q. In [NAME OF PLACE], how easy is it to get snacks like candy and chips?  Very easy…1  Somewhat easy…2  Somewhat hard…3  Very hard…4 | r. In [NAME OF PLACE], how easy is it to get sugary drinks or sodas?  Very easy…1  Somewhat easy…2  Somewhat hard…3  Very hard…4 | s. How would you rate the prices of fresh fruits and vegetables in [NAME OF PLACE]?  Very inexpensive…1  Inexpensive…2  Normal…3  Expensive…4  Very expensive…5 |
| --- | --- | --- | --- | --- | --- | --- |
|  |  |  |  |  | IF THEY ASK FOR AN EXAMPLE OF SUGARY DRINKS, SAY “ENERGY DRINKS, SPORTS DRINKS, *CHICHA MORADA* (PURPLE CORN JUICE), OR LEMONADE" |  |
| 1 |  |  |  |  |  |  |
| 2 |  |  |  |  |  |  |
| 3 |  |  |  |  |  |  |

**This is the end of the survey. Thank you very much for your participation!**
